# The influence of cognitive tasks on sensory organization test performance

**DOI:** 10.1016/j.bjorl.2020.11.009

**Published:** 2020-12-19

**Authors:** Nathan Morelli, Nicholas R. Heebner, Courtney J. DeFeo, Matthew C. Hoch

**Affiliations:** University of Kentucky, College of Health Sciences, Sports Medicine Research Institute, Lexington, United States

**Keywords:** Sensory integration, Postural control, Dual task

## Abstract

**Introduction:**

Many static postural tasks requiring vestibular contributions are completed while dual- tasking.

**Objective:**

We investigated the influence of dual-tasks on sensory integration for postural control and cognitive performance during the sensory organization test and examined the relationship between cognitive function and dual-task cost during the sensory organization test.

**Methods:**

Twenty adults completed single and dual-task versions of the six conditions of the sensory organization test were completed during two visits separated by one week. A subset of 13 participants completed three National Institute of Health (NIH)-toolbox cognitive tests including the Flanker inhibitory control and attention test, dimensional change card sort test and pattern comparison processing speed test. Wilcoxon signed rank tests were used to compare postural sway during single and dual-task sensory organization test. Friedman’s test, with pairwise comparison post-hoc tests, was used to compare single task serial subtraction performance to the 6 dual-task sensory organization test conditions. Spearman’s correlation coefficients were used to assess the relationship between cognitive performance on NIH-toolbox test and postural and cognitive dual-task cost during the sensory organization test.

**Results:**

Performing a cognitive dual-task during the sensory organization test resulted in a significant increase in postural sway during condition 1 (Z = −3.26, *p* = 0.001, ES = 0.73), condition 3 (Z = −2.53, *p* = 0.012, ES = 0.56), and condition 6 (Z = −2.02, *p* = 0.044, ES = 0.45). Subtraction performance significantly decreased in during condition 6 (Z = −2.479, *p* = 0.011, ES = 0.55) compared to single-task. The dimensional change card sort test demonstrated moderate correlations with dual-task cost of serial subtraction performance in condition 5 (dimensional change card sort test: r = −0.62, *p* = 0.02) and condition 6 (dimensional change card sort test: r = −0.56, *p* = 0.04). Pattern comparison processing speed test scores were significantly correlated with dual-task cost of postural control during condition 2.

**Conclusion:**

Performing a cognitive task during the sensory organization test resulted in significantly increased postural sway during three conditions, particularly during visual environment manipulation oppose to vestibular and somatosensory manipulation. Cognitive performance decreased during the most complex sensory organization test condition. Additionally, we found participants with poorer executive function had greater dual-task cost during more complex sensory integration demands.

## Introduction

Many activities of daily living require the simultaneous completion of a cognitive task during balancing activities which rely heavily on vestibular function, also called dual tasking. Impaired dual-task performance is associated with impaired gait in those with unilateral vestibular hypofunction, increased fall risk in geriatric and dementia patients, limited community ambulation potential, and increased musculoskeletal injury risk in athletes returning to sport following a concussion.^1^, ^2^, ^3^, ^4^, ^5^, ^6^ As a result, research integrating dual-task postural control tests has proliferated over the past decade and dual-task measures are now recommended for clinical use in many patient populations.^7^ However, there is still a lack of knowledge regarding the mechanisms underlying impaired dual-task performance.

Several theories have been proposed in efforts to explain why postural control and cognitive performance decrease during dual-task conditions. Most notable and widely researched is the capacitance sharing model.^8^, ^9^ This theory states cognitive and motor systems utilize reciprocal and overlapping parallel attentional resources to integrate information and generate functional outputs. When cognitive and motor tasks are competing for resources, such as during dual-task conditions, the functional reserve which can be devoted to either task is limited. During simple dual-tasks there are ample neural resources available to optimize and maintain motor and cognitive performance. Complex dual-tasks, requiring higher levels of neuronal activation, create competition between cognitive and motor systems. This often manifests in variable cognitive-motor interference patterns as performance of either the cognitive or motor task deteriorates.^8^ Prior studies in vestibular hypofunction during dual task conditions have supported this hypothesis.^6^, ^10^ Experimentally, the severity of cognitive-motor interference between single and dual-task conditions is quantified as dual-task cost (DTC).

One limitation regarding the capacitance sharing model warrants continued investigation. This theory claims a single source of central processing is responsible for cognitive and motor functions. This contradicts current dogma regarding the systems model of postural control and cognitive processing which arise from dynamic and adaptive coupling of different sensory and motor areas of the cortex.^11^, ^12^, ^13^ Static postural control relies on adaptive integration through reweighting and modulation of vestibular, somatosensory and visual information for sensorimotor transformations depending on the context and quality of sensory information available.^13^, ^14^, ^15^ For example, when support surfaces are unstable, healthy individuals increase the weighting of vestibular and visual inputs towards center of mass stability.^13^, ^14^ Consequently, neurophysiologic measures during postural tasks have demonstrated increased activation patterns of parietal and frontal areas with increased sensory task demands.^16^, ^17^, ^18^ Therefore, the heterogeneous nature of neural resource utilization during postural task demands suggests cognitive-motor interference and DTC may not be expressed uniformly across various sensory environments or systems.

The sensory organization test (SOT) is the current clinical gold standard for the assessment of vestibular, somatosensory and visual contributions toward center of mass stability in static stance.^13^, ^14^ Traditionally administered through computerized dynamic posturography (CDP), this test requires patients to stand on a force plate which is surrounded by a three- walled visual surrounding. Both force plate and visual surrounding are movable and systematically perturbed to alter task environments. Prior studies have investigated the effect of a secondary reaction time task on postural sway performance during the SOT.^19^, ^20^, ^21^ However, these studies have found conflicting results and used different secondary cognitive tasks. More importantly, in the time since publication a new CDP version of the SOT has been released. Instead of a three- walled surrounding, a half dome encompasses patients as a virtual environment is projected on the inside of the dome. Additionally, there are no objects in the central visual field for participants to fixate on as a stable reference sensory cue. Instead only concentric circles are projected on the temporal visual fields for sway reference. No studies have administered dual-task SOT paradigms using these technological updates which provided altered visual environments. Therefore, the environmental task demands may be substantially different and possibly result in different patterns of cognitive-motor interference.

In addition to task demands, a dearth of information exists regarding the influence of higher-order cognitive function and reserve on dual-task performance. Cognitive reserve is defined as the ability to maintain performance in the face of increased task demands, injury, or brain damage.^22^, ^23^, ^24^ It is associated with higher-order cognitive performance on tests routinely used in clinical practice and theorized to represent more complex organization of functional networks within the central nervous system (CNS) to flexibly adapt to task demands.^25^, ^26^, ^27^ Cognitive reserve also has implications for postural control, which requires complimentary explicit cortical contributions to subcortical outputs.^28^, ^29^ Therefore, according to principles of capacitance sharing model, higher-order cognitive function and reserve may play a causal role in governing the ability to adapt to dual-task demands and minimizing DTC.

Accordingly, the objective of this study was to investigate the influence of dual-tasks on vestibular, somatosensory and visual integration towards postural control and cognitive performance during the SOT. Secondarily, we aimed to examine the relationship between higher-order cognitive function and reserve on DTC and cognitive-motor interference during the SOT. We hypothesized the addition of a cognitive dual-task would result in decreased postural control and cognitive performance during conditions which impart increased sensory integration and reweighting demands. Additionally, we hypothesized those with higher clinical cognitive test performance will demonstrate reduced DTC and cognitive-motor interference. These findings will provide increased insight into DTC patterns in various sensory states and elucidate possible mechanisms underlying the ability to mitigate DTC.

## Methods

### Participants

Twenty adults (12 females, 8 males; age: 21.9 ± 3.8 years; height: 1.70 ± 0.01 m; weight: 69.4 ± 15.0 kg) volunteered in this study. Participants were excluded if they were currently being treated for a lower extremity injury, self-reported ADD or ADHD, were taking medications which may influence cognition or balance, or had sustained a concussion within the past 6-months. Prior to testing, each subject provided written informed consent which was approved by the institutional review board and ethics committee (approval number 52918).

### Procedures

Participants completed single and dual-task assessments during two data collections separated by 1 week. Prior research on the test-retest reliability of the SOT, in a similar population, has revealed a learning effect between the first and second test.^30^ To address this specific concern single and dual-task version of the SOT were counterbalanced between days for the entire cohort. Therefore, 10 subjects completed the single-task SOT on the first visit and 10 subjects completed the dual-task SOT during the first visit. Additionally, conditions 2–6 of the SOT were randomized for each subject to mitigate practice effects. The investigators did not direct the participants to focus attention on the postural or cognitive task during dual-task conditions. All participants were secured to a safety harness throughout the entire test.

The 6 conditions of the SOT were administered using Bertec®’s computerized dynamic posturography (CDP) (Bertec Corporation, Columbus, OH). The conditions comprised of: C1: eyes open, fixed surface and visual surrounding; C2: eyes closed, fixed surface; C3: eyes open, fixed surface and oscillating visual surrounding; C4: eyes open, oscillating surface and fixed visual surrounding; C5: eyes closed, oscillating surface; C6: eyes open, oscillating surface and visual surrounding.^31^ Participants were alerted to the condition manipulations prior to initiating the first trial. Each condition lasted 20 seconds and was completed three times. Base of support was normalized for each subject to approximately shoulder width apart. If a subject fell or altered their base of support during the trial the score was discarded, and a member of the research team repositioned the subject’s feet back to the correct position. A unique feature of the Bertec® CDP is that it projects a virtual visual background onto an immersive dome structure for visual sway reference. The laboratory was set to 45 ± 5l × using a digital Leaton Luxmeter (Shenzhen DeXi Electronics Co., Ltd, Shenzhen, China) to ensure consistent illumination of the projected background.

For each trial, an “equilibrium score” is provided by the Bertec® Balance Advantage™ software and is calculated as the ratio between the anterior-posterior peak-to-peak sway during each trial to the theoretical center of gravity limits of stability (12.5°).^32^ Equilibrium scores are reported on an interval scale with 0 representing a fall or stopped trial and 100 indicating little to no sway. Scores were averaged for each trial for final reporting. Sensory ratios were calculated by Bertec Balance Advantage software for somatosensory (C2:C1), vestibular (C5:C1), and visual (C4:C1) systems.

Serial subtraction was utilized as the cognitive dual task for this study. Participants were given a random 2-digit number between 99 and 80 and asked to subtract by 6 s or 7 s. For single-task performance, participants sat in a chair in a quiet room and assessed over a 20-second period. For dual-task versions of the SOT, participants were given a new set of numbers for each condition while their performance was recorded. Cognitive tasks were standardized across subjects, so each participant completed the same serial subtraction task per test condition. Overall performance was calculated by subtracting the sum of incorrect responses from the sum of correct responses.

The DTC of postural and cognitive performance was calculated for each condition of the SOT using the following equation: DTC = [(dual-task performance − single task performance)/single task performance] × 100. Higher serial subtraction and equilibrium score DTC signifies improved performance during dual-task conditions.

For the secondary objective, a subset of 13 participants completed multiple cognitive assessments from the NIH Toolbox® (NIHTB) Cognitive Battery which has demonstrated excellent reliability and construct validity.^33^ We selected tests which assessed attention, spatial processing, cognitive flexibility, executive functioning, and processing speed. Specifically, participants completed the Flanker inhibitory control and attention test (FICA), dimensional change card sort test (DCCS), and pattern comparison processing speed test (PCPS) on a 10.2 in iPad (iPad 5th Generation, Apple, Cupertino, CA) (Table 1). Participants completed these tests on the first visit while seated a table in a quiet room. Fully corrected scores, accounting for age, sex, education level, race, and ethnicity, were calculated in the NIHTB software. A composite score was calculated for each participant from the sum total of fully corrected scores of all three NIHTB tests.

**Table 1:** NIHTB test descriptions.

Test	Cognitive domain(s) assessed	Description
Flanker Inhibitory Control and Attention Test (FICA)	Executive function; inhibitory control and attention	A central arrow facing left or right is surrounded by arrows on either side. The surrounding arrows are pointing the same or opposite direction as the central arrow. Individuals must identify the direction the central arrow is pointing as quickly as possible.
Dimensional Change Card Sort Test (DCCS)	Executive function; cognitive flexibility	Two different objects, different in shape and color, are presented side-by-side. A third object is presented in the center of the screen. Individuals must to match either the shape or color of the two objects to a third object as quickly as possible.
Pattern Comparison Processing Speed Test (PCPS)	Cognitive processing speed	This assessment requires individuals to indicate if two objects are the same or different as quickly as possible.

### Statistical analysis

Descriptive statistics (median and interquartile range) were calculated for equilibrium scores and serial subtraction performance during single and dual-task conditions. Normality of included variables was assessed using the Shapiro-Wilk test. A Freidman’s test with Wilcoxon Signed Rank tests were used to determine the influence of cognitive dual-tasks on postural sway performance (i.e. equilibrium scores) for each condition of the SOT as well as sensory ratios. The influence of task conditions on serial subtraction performance was assessed using Wilcoxon Signed Rank tests between single task performance and each dual-task SOT condition. Spearman’s correlation coefficients were used to assess the relationship between cognitive performance on NIHTB test and postural and cognitive DTC during the SOT. Non-parametric effect sizes (ES) were calculated for postural sway and cognitive performance between single and dual-task conditions. Effect sizes were interpreted as small: 0.2, medium: 0.5 and large: 0.8.^34^ Correlation coefficients were interpreted as little to no relationship: ≤0.25, fair: 0.26–0.50, moderate to good: 0.51–0.75, and good to excellent: >0.75.^35^ The level of significance for all analyses was set α-priori at p ≤ 0.05. All statistics were completed in SPSS version 25 (IBM, Armonk, NH).

## Results

Performing a cognitive dual-task during the SOT resulted in a significant decrease in equilibrium scores during C1 (Z = −3.26, *p* =  0.001, ES = 0.73), C3 (Z = −2.53, *p* = 0.012, ES = 0.56), and C6 (Z = −2.02, *p* = 0.044, ES = 0.45) (Table 2 for detailed results). A significant Freidman’s test (*p* = 0.038) with Wilcoxon Signed Rank post-hoc testing revealed a significant decrease in serial subtraction performance during C6 (Z = −2.48, *p* = 0.013, ES = 0.55) compared to single-task performance (Table 3).

**Table 2:** Postural control performance during single and dual-task SOT conditions (Median [IQR]).

SOT condition	Single-task	Dual-task	p-value	Effect size
C1	92.7 (2.2)	89.8 (6.2)	0.001	0.73
C2	90.7 (4.2)	89.8 (8.2)	0.064	0.41
C3	91.3 (4.9)	88.3 (7.42)	0.012	0.56
C4	76.0 (13.3)	76.8 (17.8)	0.322	0.22
C5	63.0 (10.3)	64.2 (17.13)	0.467	0.16
C6	65.3 (15.5)	60.0 (21.7)	0.044	0.45
Somatosensory	98.5 (3.72)	100.0 (6.5)	0.407	0.19
Visual	82.0 (9.75)	83.0 (21.75)	0.602	0.12
Vestibular	67.5 (11.5)	74.0 (29)	0.421	0.18
Composite score	75.6 (11.2)	75 (14.0)	0.286	0.24

**Table 3:** Serial subtraction performance between single and dual-task conditions (Median [IQR]), (significance and effect sizes represent comparison to ST).

	Serial subtraction performance	p-value	Effect size
Single task	7.5 (5.75)		
Dual-task SOT condition			
C1	5.83 (5.00)	0.167	0.31
C2	5.67 (5.77)	0.126	0.34
C3	5.67 (6.17)	0.13	0.34
C4	6.00 (4.25)	0.083	0.38
C5	5.67 (4.83)	0.145	0.33
C6	5.33 (2.83)	0.013	0.55

**Figure 1:**
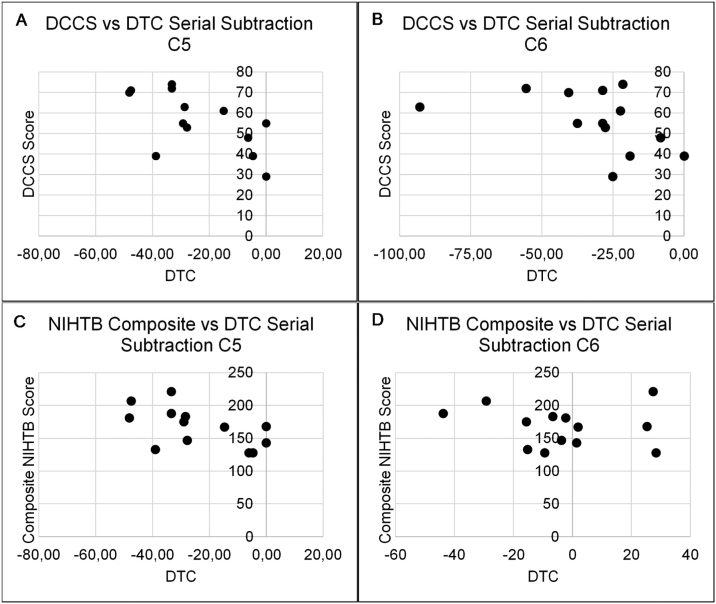
DTC of serial subtraction performance was significantly associated with Dimensional Change Card Sort (DCCS) test performance in C5 (A) and C6 (B) of the SOT. Additionally, composite NIHTB scores demonstrated significant correlations with C5 (C) and C6 (C) of the SOT.

The relationship between higher-order cognitive function and cognitive reserve to DTC of postural and serial subtraction performance during the SOT are presented in Table 4, Table 5. There was only one significant association between NIHTB tests and DTC of equilibrium scores during the SOT. Specifically, PCPS to DTC of equilibrium score in C2 (r = 0.55, *p* = 0.05). Additionally, the DCCS and composite NIHTB fully corrected scores demonstrated moderate to good correlations with DTC of serial subtraction performance in C5 (DCCS: r = −0.62, *p* =  0.02; composite NIHTB: r = −0.59, *p* = 0.03) and C6 (DCCS: r = −0.56, *p* = 0.04; composite NIHTB: r = −0.66, *p* = 0.01) (Fig. 1).

**Table 4:** Spearman’s correlations between NIHTB measures and DTC of SOT serial subtraction performance.

SOT condition	Dimensional change card sort test	Flanker inhibitory control and attention test	Pattern comparison processing speed test	Composite NIHTB
C1	0.05	−0.01	−0.32	−0.10
C2	−0.14	−0.20	0.01	−0.15
C3	−0.03	−0.10	−0.13	0.01
C4	−0.30	−0.36	−0.51	−0.35
C5	−0.62^a^	−0.25	−0.40	−0.59^a^
C6	−0.56^a^	−0.50	−0.50	−0.66^a^

a *p* < 0.05.

**Table 5:** Spearman’s correlations between NIHTB measures and DTC of SOT equilibrium scores.

SOT condition	Dimensional change card sort test	Flanker inhibitory control and attention test	Pattern comparison processing speed test	Composite NIHTB
C1	0.04	0.43	0.08	0.10
C2	0.10	0.31	0.55^a^	0.33
C3	0.22	0.16	0.31	0.28
C4	−0.46	−0.35	−0.04	−0.30
C5	−0.46	−0.25	−0.11	−0.37
C6	−0.13	−0.15	−0.08	−0.24

## Discussion

The primary aim of this study was to explore the influence of dual-tasks on postural control and cognitive performance during various sensory states of the SOT, administered through Bertec® CDP. We found that performing a serial subtraction task resulted in significantly increased postural sway during three conditions and decreased cognitive performance during C6 of the SOT. The secondary aim of this study was to investigate the relationship between higher-order cognitive function and reserve on DTC during the SOT. Contrary to our hypothesis, results suggest increased higher-order cognitive function, including executive function, attention, processing speed, and conflict resolution, are associated with greater DTC of cognitive performance during dual-task conditions. Importantly, these relationships were only present in conditions requiring significant sensory integration demands to somatosensory and visual systems. Our results provide supplemental support to prior investigations of task complexity on dual-task performance and novel insights into the interaction between higher-order cognitive function and DTC.

The findings of this study are similar to prior investigations of dual-task performance during different static postural control sensory states. With regards to the SOT, Lanzarin et al.^21^ found decreased postural control during the concurrent completion of mathematics tasks in C1 and C6. Remaud et al.^36^ found postural sway increased significantly only during dual-task Balance Error Scoring System conditions with challenging somatosensory and visual system integration and reweighting demands. In order to generate appropriate postural motor commands sensory information must be integrated and salient stimuli used to guide responses must be identified. These findings suggest the simultaneous completion of a cognitive task interfere with this process, limiting the ability to identify sensory information needed to guiding postural control during conditions which manipulate or perturb visual and somatosensory information.

Our findings differ slightly from Lanzarin et al.^21^ as we found significantly decreased equilibrium scores in C3. It’s possible the virtual visual field sway manipulation induces greater sensory reweighting demands compared to the Neurocom® CDP. If so, the overall task demands may have been higher in our paradigm, surpassing dual-task processing thresholds, resulting in decreased postural control. This could provide partial explanation for the moderate effect sizes seen in C3 and C6 of this study. Moreover, this would support prior investigations of deleterious postural sway during conditions which impart increased somatosensory and visual integration and reweighting demands.^36^, ^37^, ^38^ Conditions which require the highest contribution of the vestibular system, C5 and C6, only demonstrate partial deterioration during dual-tasking. Taken together, these data provide additional evidence of the deleterious nature of somatosensory and visual sensory manipulation toward cognitive-motor interference.

Interestingly, C1 exhibited significant single and dual-task differences in prior studies^19^, ^21^ and demonstrated larger effects than C3 and C6 in this current protocol. These findings are antithetical to concepts of capacitance sharing model as C1 offers the least difficult task demands. The significant differences in performance during C1 may therefore be a result of task novelty as it was always completed first during data collection in this and previous studies. This could influence not only overall task performance, but also alter attention prioritization which is known to impact DTC.^8^

Serial subtraction performance during the SOT experienced a significant decrease only during postural conditions which perturbed somatosensory and visual sensory information (i.e. C6). Interestingly, cognitive performance demonstrated small effect sizes during other SOT conditions with significant postural performance decreases (i.e. C1 and C3). These data suggest that when cognitive task difficulty is held constant, there may be a critical threshold of postural task complexity before cognitive function also experiences significant deterioration. These findings are similar to Resch et al.^19^ and Remaud et al.^36^ who also found the largest deterioration of secondary cognitive task performance during conditions which manipulated both visual and somatosensory environments. Additionally, since no direction was given to focus attention on either the postural or cognitive task, these findings could represent a salient relationship of implicit cognitive-motor interactions. Namely, when no explicit direction is given, cognitive performance may be preserved at lower sensory integration difficulties even in the presence of postural control deterioration. Significant cognitive performance deterioration may only occur during exceedingly complex sensory demands or when failure to divert explicit resources towards postural motor responses could result in loss of balance.

The performance decrements demonstrated in this study offer partial support to the capacitance sharing model. Neurophysiologic recordings during static postural control have found cortical activity of areas associated with integrating somatosensory and visual information to increase with task complexity.^17^, ^18^ Furthermore, these regions have demonstrated increased activity during dual-tasking.^39^ The increased postural sway during dual-task conditions which manipulated the virtual environment (i.e. C3 and C6) suggests cognitive tasks limit the ability to integrate and reweight visual information. While C6 also perturbs somatosensory information, a result of the unstable surface, the minimal difference in effect size between C3 and C6 suggests cognitive dual-tasks have little influence on somatosensory integration and reweighting. Therefore, the heterogeneous nature of cognitive-motor interference noted during the SOT suggests capacitance sharing model dual-task processing limitations are not expressed uniformly across various sensory environments.

Dual-task performance has been predicated on the functional integrity and adaptive capacity of executive function, attention, working memory, and response selection systems.^37^, ^40^ According to principles of capacitance sharing model, higher-order cognitive function and reserve may play a causal role in governing the ability to modulate DTC. A recent study of collegiate athletes who completed the modified Clinical Test of Sensory Interaction on Balance, a clinical surrogate of the SOT, found weak correlations (r < −0.20, *p* < 0.05) between Immediate Post-Concussion Assessment and Cognitive Test visual motor speed and dual-task vestibular ratios (eyes closed, foam surface: eyes open, firm surface).^41^ However, direct comparison to dual-task performance is inherently flawed as this analysis fails to account for the subject’s ability to adapt to complex task demands relative to single-task performance. The only NIHTB measure demonstrating significant association to postural control DTC was Pattern Comparison Processing Speed to C2 (r = 0.55, *p* = 0.05). Contrary to our hypothesis, higher composite NIHTB and DCCS scores were associated with larger negative DTC of cognitive performance during C5 and C6. This relationship opposes those seen in older adults, Parkinson’s disease and multiple sclerosis.^42^, ^43^, ^44^ Nonetheless, the association between higher-order cognitive function and reserve and DTC may be functionally significant, as these relationships were only seen in the two most difficult conditions of the SOT.

Future implementation of this test into pathologic patient populations could improve the clinical detection of dual-task deficits and elucidate possible sensory integration impairments. CNS injury and atrophy could reduce the functional capacity to optimize dual-task performance, the consequences of which may not only manifest as larger DTC, but also result in DTC at lower thresholds of task complexity. Additionally, visual field perturbations during static stance have elicited increased sway in pathologies which either injure areas of the cortex responsible for integrating multimodal sensory information (e.g. traumatic brain injury), or which create over- reliance on visual information for postural control (e.g. Parkinson’s disease).^45^, ^46^ Dual-task SOT paradigms using the virtual visual field surrounding of the Bertec® CDP could increase test sensitivity to detecting these impairments. Additionally, the continued investigation into the role of higher-order cognitive function and reserve to DTC could provide further insights into the ability to maintain dual-task performance across various postural conditions.

There are several limitations to this study. There was a small sample size of subjects included in both of the aims of this study. While the effect sizes and correlations found in this study were robust, this nonetheless minimizes the certainty of our conclusion. We attempted to mitigate practice effects by randomizing C2–C6. However, all participants completed C1 first and did not take practice trials prior to beginning testing. This could account for the significant differences in performance during C1 seen here and in prior investigations. Additionally, cognitive task performance during dual-tasks was assessed using number of correct and incorrect responses. This method has been used in prior investigation of dual-task postural control paradigms.^41^, ^47^ A more sensitive measure of performance, such as reaction time, could be more sensitive to detecting DTC.

## Conclusion

This study expands on prior investigations of cognitive-motor interference in healthy adults during various postural task demands. Performing a cognitive task during the SOT resulted in significantly increased postural sway during three conditions and significantly decreased serial subtraction performance during C6. These results provide supplemental insight into capacitance sharing model of dual-task performance deterioration during tasks which manipulate somatosensory and visual sensory information. Additionally, we found cognitive function was negatively associated with DTC during more complex sensory integration demands (i.e. C5 and C6). These data provide novel insight into possible mechanisms regulating the ability to modulate DTC during various postural task demands.

## Conflicts of interest

The authors declare no conflicts of interest.
